# The Changing Face of Dystonia—Enhancing Diagnosis by Moving Beyond Traditional Clinical Phenotyping

**DOI:** 10.1002/mdc3.70189

**Published:** 2025-06-26

**Authors:** Niroshan Jeyakumar, Kishore R. Kumar

**Affiliations:** ^1^ Movement Disorders Unit, Westmead Hospital, Department of Neurology Westmead Hospital Westmead NSW Australia; ^2^ Sydney Medical School University of Sydney Camperdown NSW Australia; ^3^ Molecular Medicine Laboratory and Neurology Department Concord Repatriation General Hospital Concord NSW Australia; ^4^ Translational Neurogenomics Group, Genomic and Inherited Disease Program The Garvan Institute of Medical Research Darlinghurst NSW Australia; ^5^ School of Clinical Medicine, Faculty of Medicine and Health, St Vincent's Healthcare Clinical Campus University of New South Wales Darlinghurst NSW Australia

**Keywords:** dystonia, next generation sequencing, long‐read sequencing, wearable, machine learning

## Dystonia as a Concept

The latest definition of dystonia comes from Albanese et al's 2013 *Phenomenology and Classification of Dystonia: A Consensus Update*, now over a decade old.^1^ Dystonia is defined by the expert international consensus committee as “sustained or intermittent muscle contractions causing abnormal, often repetitive, movements, postures or both,” with suggestive features including initiation or exacerbation by voluntary action and overflow muscle activation. Classification is made along two axes: (i) clinical characteristics, which includes age of onset, body distribution, temporal pattern and associated features, and (ii) etiology, sub‐classified as degenerative, static/structural or neither and inherited, acquired or idiopathic. The foundations of former definitions remain recognizable in this update, even dating back to Oppenheim's first account of “dystonia musculorum deformans” in 1911 where “muscle tone was hypotonic at one occasion and in tonic muscle spasm at another, usually, but not exclusively, elicited upon voluntary movements,” supporting dystonia's position in movement disorders lore as a true, distinct entity with consistent properties and not merely a nosological construct.^2^

Despite this, a clear understanding of the underlying pathophysiology of dystonia has remained elusive. Some themes, such as disruption of basal ganglia–cerebello‐thalamo‐cortical circuits, disordered sensorimotor integration, alterations in synaptic plasticity and dopaminergic dysfunction have emerged but for the most part neuropathological studies have failed to show overt micro‐ or macroscopic abnormalities.^3^ Consequently, there is no reliable biomarker, making the diagnosis of dystonia challenging and reliant on aforementioned clinical criteria.

The limitations of the current approach to dystonia are exemplified by: (i) long delays between symptom onset and diagnosis, ranging between 4 and 10 years depending on subtype and (ii) poor inter‐rater reliability with low levels of agreement for the diagnosis of different subtypes of dystonia among neurologists with different levels of training.^4^ The inherently variable nature of dystonia over time complicates matters further as usual clinical rating scales become difficult to interpret. All of this drives the need for incorporating new technologies to enhance the assessment of dystonia and advances in areas like genetics, wearables and artificial intelligence are paving a way forward.

## Advances in Genetic Testing for Dystonia

In the absence of a general biomarker for dystonia, the identification of a causative gene variant can be extremely helpful, providing diagnostic clarity as well as informing treatment options. For instance, Segawa syndrome or *GCH1‐*associated dopa‐responsive dystonia (DRD), is exquisitely responsive to low doses of levodopa, copper chelation therapy can improve the dystonia associated with Wilson's disease caused by biallelic variants in *ATP7B*, and deep brain stimulation can be highly effective in *TOR1A*‐associated dystonia.^5^ However, since the discovery of *GCH1* in 1994, the first monogenic cause of dystonia to be identified, over 50 others have been reported and many more still causing combined syndromes.^6^ Whilst some have distinctive phenotypes (eg, DYT‐*SGCE* causing myoclonus‐dystonia), considerable phenotypic overlap exists in other cases (eg, DYT‐*TOR1A* and DYT‐*KMT2B*), making a purely phenotype‐driven testing approach fraught with danger. Moreover, with the sheer number of new genes being identified, the era of the clinical expert able to keep personal inventory of all genotype–phenotype correlations and rely on experience and pattern recognition alone may be reaching the limits of practicality. What then is the way forward?

Modern genetic testing technologies may be part of the answer. The advent of next‐generation sequencing (NGS) in the early 2000s revolutionized genetic testing by allowing massively parallel sequencing of millions of DNA fragments simultaneously, leading to a dramatic increase in throughput compared to traditional Sanger sequencing. NGS can be applied to dystonia panels to sequence all known dystonia‐associated genes at once or, increasingly, can even be used to sequence the whole exome, known as whole exome sequencing (WES), which has the advantage of being able to detect novel genes not previously associated with dystonia and permit reanalysis of data in the future. WES is particularly helpful for detecting conditions that typically present with phenotypes other than dystonia and so may not be included in routine, pre‐selected dystonia gene panels such as ataxia‐telangiectasia, caused by variants in *ATM*, where late‐onset disease can present as isolated dystonia without ataxia.^7^

Whole genome sequencing (WGS) is more powerful still as it can additionally detect disease‐causing variants that are not captured on WES. For example, WGS was able to detect a paired‐deletion inversion complex structural variant in the *GCH1* gene of a patient with a clinical phenotype of DRD despite traditional single gene sequencing techniques failing to detect the disease‐causing variant.^8^ Here, we see that unlike the opposite problem alluded to earlier, the issue may not just lie in the clinician being unable to recognize a complex phenotype but also in the limitations of routine tests for detecting complex genotypes.

This case also highlights the broader concept of the utility of re‐analyzing genomic data. In fact, it has been shown that in dystonia specifically, systematic re‐analysis of genomic data using an updated gene panel together with gene discovery collaborative efforts, even within as little as 5 years on from initial testing, can increase diagnostic yield.^9^ It is likely to increase even further with emerging technologies like long‐read sequencing offering further critical improvements over NGS, in particular in the detection of repeat expansions, structural variants, copy number variants and methylation status that may be missed with short‐read techniques.^10^, ^11^, ^12^

## Wearable Devices Bringing the Clinic to the Home

Whilst modern gene sequencing technologies hold great promise for the diagnosis of dystonia when there is early‐onset disease or a strongly positive family history that point to a monogenic cause, a large majority of cases do not fit into these categories and may be complex polygenic, acquired or idiopathic. In such cases, accurate phenotyping remains essential and yet at the same time difficult.

Clinical neurophysiological tests such as surface electromyography can help, with co‐contraction of agonist and antagonist muscles and overflow of activity to inappropriate muscles during voluntary movement being characteristic features of dystonia.^13^ However, the sensitivities and specificities of these tests have not been well established with no universally accepted standardized criteria and differentiating dystonia from mimics like chorea and psychogenic movement disorders can still be challenging. Furthermore, access to specialized laboratories offering testing may vary by region and the reliability of testing may be influenced by the dynamic nature of dystonia, which can be task‐specific in ways difficult to replicate in a laboratory setting and vary over time.

Wearable sensor technologies have emerged as a potential solution to some of these practical limitations. These utilize inertial measurement units that combine accelerometers, gyroscopes and/or magnetometers to quantify motion parameters like linear acceleration, angular velocity and orientation, respectively.^14^ Their small size and comfort allow them to be worn for extended durations by users in their home environments, making them attractive for the monitoring of movement disorders. This is particularly valuable in dystonia to capture the variability in the nature and severity of the movements over time, providing a truer representation of the condition than a single cross‐sectional assessment and therefore potentially improving diagnostic accuracy. It also allows the objective tracking of treatment responses to better inform management decisions compared to sole reliance on patient‐reported histories, which are prone to gaps and biases.

Most existing studies on wearable devices have focused on Parkinson's disease, however, cervical dystonia has received some recent attention,^15^, ^16^ as well as dystonia associated with cerebral palsy^17^, ^18^ and X‐linked dystonia‐parkinsonism.^19^ These have so far only been small‐scale “proof‐of‐concept” studies of kinematic data in comparison to healthy controls or clinical rating scales but more rigorous validation protocols perhaps against conventional neurophysiological testing are needed before wearable devices can truly direct diagnosis and management.

## The Role of Artificial Intelligence and Machine Learning

The large amounts of data obtained by extended monitoring with wearable sensors also presents the perfect opportunity for training machine learning models to recognize dystonia. For example, Parisi et al applied a random forest model to analyze wearable sensor data from participants with X‐linked dystonia‐parkinsonism and achieved accuracies of greater than 90% in some instances for detecting dystonia in comparison to clinician ratings.^19^ Such machine learning‐based classifications are desirable because they are algorithmic, bringing standardization and therefore greater objectivity and reliability to assessment than traditional clinical phenotyping and clinical interpretation of neurophysiology. Harnessing new developments in the underlying mathematical foundations of these models can improve their classification accuracies further, for instance, via robust optimization approaches that can handle noise in input data, ubiquitous in real‐life clinical scenarios.^20^, ^21^

Whilst classical machine learning models like random forests perform well on simple data like kinematic parameters, they struggle with more complex data of higher dimensionality and are reliant on domain expertise to predefine features for analysis (eg, stride length and step height as features of gait). More sophisticated, so‐called unsupervised “deep learning” models can overcome these limitations too, providing novel insights unconstrained by preconceived notions and theories. For example, Valeriani et al recently developed a deep learning platform, DystoniaNet, capable of distinguishing participants with dystonia from healthy controls with 98.8% accuracy simply by looking at raw, structural brain MRIs.^22^ The model was able to identify a distinct microstructural neural network biomarker of dystonia involving various brain regions such as the corpus callosum, thalamic radiations, inferior fronto‐occipital fasciculus and inferior temporal and superior orbital gyri. Such regions would have been likely impossible to predefine otherwise at a time when many once “canonical” pathophysiological findings in dystonia are being overturned by recent evidence pointing to dysfunction of a much more complex, highly interconnected network than currently understood.^23^

## How the Future Might Look

**Figure 1:**
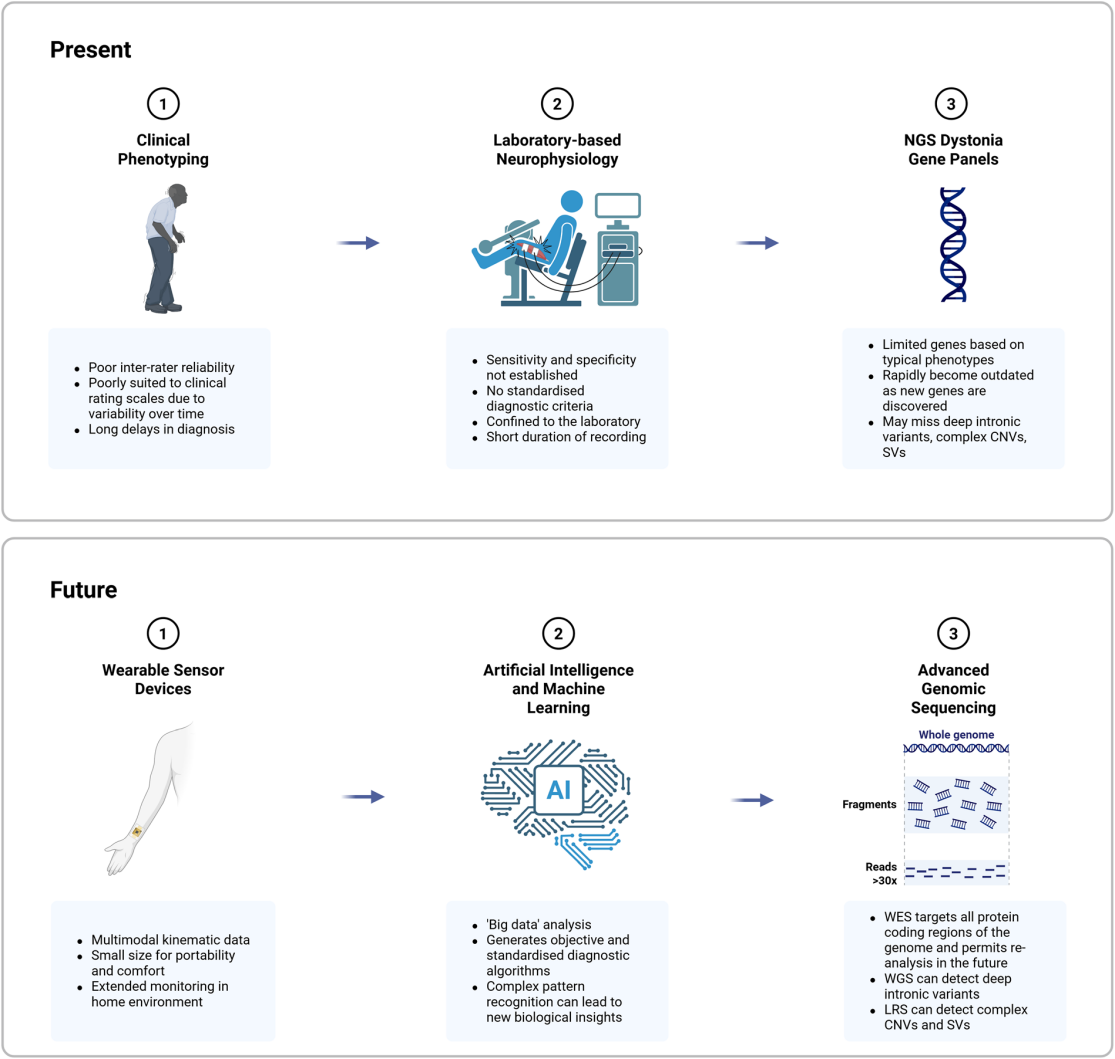
Diagnostic pathways for dystonia: present and future. NGS, next‐generation sequencing; CNV, copy number variant; SV, structural variant; WES, whole exome sequencing; WGS, whole genome sequencing; LRS, long‐read sequencing. Created in BioRender.

We have come full circle, to an era where modern technologies may themselves resolve the original fundamental pathophysiological gaps they were created to circumvent. It does not seem so inconceivable that patients might one day own personal wearable devices that will capture their motion data for input into an artificially intelligent platform to determine their precise subtype of dystonia, linked to a particular genetic variant, confirmed by a specific, highly accurate gene sequencing test (Fig. 1). The next frontier will be developing machine learning models that can recognize combined syndromes such as dystonia‐parkinsonism and myoclonus‐dystonia and indeed with deep learning techniques we may no longer need to be confined to these classical, categorial phenomenological descriptions at all. This future may be fast approaching. What does this mean for the movement disorders neurologist? We, too, will need to evolve. The modern movement disorders neurologist will need to be much more than just a clinician but also fluent in the language of the geneticists, the engineers and the computer scientists to ride the growing wave of modern technological expansion towards the promised land of more efficient diagnosis and personalized treatment. Else, we risk drowning beneath it.

## Author Roles

(1) Research project: A. Conception, B. Organization, C. Execution; (2) Statistical Analysis: A. Design, B. Execution, C. Review and Critique; (3) Manuscript Preparation: A. Writing of the first draft, B. Review and Critique.

N.J.: 1A, 1B, 1C, 3A, 3B.

K.R.K.: 1A, 1B, 1C, 3B.

## Disclosures

**Ethical Compliance Statement:** The authors confirm that the approval of an institutional review board was not required for this work. Informed patient consent was not necessary for this work. We confirm that we have read the Journal's position on issues involved in ethical publication and affirm that this work is consistent with those guidelines.

**Funding Sources and Conflict of Interest:** No specific funding was received for this work. The authors declare that there are no conflicts of interest relevant to this work.

**Financial Disclosures for the previous 12 months:** KRK is supported by research funding from the Ainsworth 4 Dystonia Genetic Research Mission, Medical Research Future Fund and Lord Mayors Charitable Trust. KRK receives honorarium from The International Parkinson and Movement Disorder Society and The Limbic. NJ declares that there are no additional disclosures to report.

## Data Availability

Data sharing not applicable to this article as no datasets were generated or analysed during the current study.
